# 

**Published:** 1897-12

**Authors:** 


### Society Notes.

**East Texas Medical Association.**—The next semi-annual meeting of the East Texas Medical Association will be held at Troupe, Texas, June 11th, 1898. Every doctor in Texas is cordially invited to be present and contribute a paper or report interesting cases.

We are assured by the president, Dr. W. S. Lacy, that this will be the most successful meeting in the history of the association, all of which is doubly guaranteed by the well known hospitality of the people at Troupe, and the East Texas doctors. Send titles of papers, etc., to the secretary, E. C. Foster, M. D., Fruit, Texas.

### South Texas Medical Association.

The third semi-annual meeting of the South Texas Medical Association will be held in the Elks Hall, Beaumont, Texas, on Tuesday, December 28th, 1897. The following interesting program has been prepared:

Meeting called to order at 9:30 a. m., by the president, Dr. Bat Smith, of Wharton.

Invocation—Rev. W. W. Watts.

Address of welcome—Hon. Geo. C. O'Brien.

Response for Association—Dr. Joseph A. Mullen, of Houston.

"The Nose, its Functional Integrity an Important Factor in the Health of its Possessor"—Dr. E. S. Heisig, of Houston. Discussion opened by Drs. Vard Hulen, of Galveston, and J. W. Cruse, of Beaumont.

"Intestinal Sepsis, the Antiseptic Treatment,"—Dr. F. B. King, of Houston. Discussion opened by Drs. S. C. Red, of Houston, and A. H. Schenk, of Kinney.

"A New Operation for Piles"—Dr. Wm. Keiller, of Galveston. Discussion opened by Drs. J. E. Thompson, of Galveston, and J. O. Williams, of Houston.

"Report of Six Cases of Puerperal Eclampsia, with three Deaths"—Dr. J. S. Price, of Beaumont. Discussion opened by Drs. J. T. Y. Payne, of Galveston, and S. W. Sholars, of Orange.

"The Diagnosis of Malaria"—Dr. H. H. Forline, of Houston. Discussion opened by Drs. H. A. West, of Galveston, and W. J. Bleivett, of Beaumont.

"Anatomical Changes in Malarial and Yellow Fevers"—Dr. Bat Smith, of Wharton. Discussion opened by Drs. Wm. Keiller, of Galveston, and J. E. Cannon, of Alvin.

"Influenza with Special Reference to the Genito-Urinary Organs"—Dr. O. L. Norsworthy, of Houston. Discussion opened by Drs. W. W. Cunningham, of Beaumont, and J. Saunders, of Orange.

"The Operative Treatment of Hairlip"—Dr. J. E. Thompson, of Galveston. Discussion opened by Drs. R. T. Morris, of Houston, and Bat Smith, of Wharton.

"Uterine Curettage"—Dr. J. W. Scott, of Houston. Discussion opened by Drs. S. C. Red, of Houston, and B. F. Calhoun, of Beaumont.

"Dengue and Allied Fevers"—Dr. R. T. Morris, of Houston. Discussion opened by Drs. J. W. Scott, of Houston, and Geo. H. Lee, of Galveston.

"Why so Many Young People Wear Glasses Today"—Dr. Vard Hulen, of Gålveston. Discussion opened by Drs. Joseph A. Mullen, and E. S. Heisig, of Houston.

"Operative Gynecology, With a Report of Cases"—Dr. T. L. Kennedy, of Galveston. Discussion opened by Drs. R. W. Knox, and Donald McKay, of Houston.

"Epestaxis as a Symptomatic Complication of the late Dengue Epidemic"—Dr. Joseph A. Mullen, of Houston. Discussion opened by Drs. Hulen and T. L. Kennedy, of Houston.

"Nature and Art in the Cure of Disease"—Dr. B. F. Calhoun, of Beaumont. Discussion opened by Drs. F. B. King, of Houston, and J. F. Collier, of Conroe.

